# Dynamic changes in the hindgut bacterial community of pre-weaning crossbred beef calves in a pasture-based system

**DOI:** 10.1128/spectrum.01730-26

**Published:** 2026-08-21

**Authors:** A. Post, T. Webb, N. Indugu, B. I. Smith, D. W. Pitta

**Affiliations:** 1 Department of Clinical Studies, New Bolton Center, University of Pennsylvania, School of Veterinary Medicine 189482, Kennett Square, Pennsylvania, USA; The University of Tennessee Southern, Pulaski, Tennessee, USA

**Keywords:** beef calf microbiome, pasture-based systems, maternal influence, early-life microbial colonization, microbiome–environment interactions, extensive systems, angus calves, South Poll calves

## Abstract

The early-life gut microbiome represents a window during which microbial alterations may impact long-term health and productivity. However, most research has focused on confined dairy systems with limited breed variation. To address this gap, we examined bacterial community dynamics in pasture-raised South Poll × Angus and Angus cow–calf pairs (*n* = 7). Fecal samples were collected from calves at birth and weekly through 8 weeks, with dam’s samples collected post-calving. DNA was extracted from fecal samples, and bacterial communities were profiled using the V1–V2 region of the 16S rRNA gene. Alpha diversity increased significantly with age (richness *ρ* < 0.01, Shannon *ρ* < 0.01), while beta diversity exhibited strong temporal structuring (weighted *R*² = 0.33, *ρ* = 0.001; unweighted *R*² = 0.38, *ρ* = 0.001), as calf communities converged toward adult reference states by 8 weeks. Sex exerted modest but detectable effects on community structure. Taxonomic analyses identified a core microbiome of 25 taxa that accounted for most community structure and defined discrete temporal phases of development. Early fecal samples were dominated by *Lactobacillus*, *Bacteroides*, and facultative anaerobes. These taxa declined rapidly with age, coincident with increases in *Ruminococcus*, *Blautia*, *Dorea*, *Lachnospiraceae*, and *Clostridiales* (*q* < 0.05). Calf average daily gain was positively correlated with *Streptococcus*, *Dorea*, and *Bacillus* and negatively correlated with *Ruminococcaceae* (*ρ* > 0.3, *q* < 0.05). These findings demonstrate rapid gut microbiome development in pastured beef calves, similar in pace but distinct in composition from confined dairy systems, underscoring the importance of environment and breed diversity in shaping early-life microbial colonization.

**IMPORTANCE:**

Early-life microbial colonization plays a critical role in shaping gastrointestinal physiology and immune-cell maturation, with impacts on long-term productivity in cattle. However, most studies describing microbial succession focused on Holstein calves raised in confinement systems, leaving the microbial ecology of beef calves, particularly crossbreds and those on pasture, poorly characterized. In this study, we longitudinally characterized hindgut microbial development in Angus × South Poll and Angus calves during the first 8 weeks of life. We demonstrate a structured microbial succession driven by a core set of taxa that evolves with age, with continuous dominance of *Lactobacillus*. These findings establish foundational knowledge of microbiome assembly in a breed of beef calves growing in popularity and highlight microbial taxa and community structures that may relate to breed genetics influencing growth and health. Understanding microbial development in pasture-based beef systems provides an essential framework for designing microbiome-informed management or nutritional interventions aimed at improving productivity and sustainability, particularly in pasture-raised animals.

## INTRODUCTION

The gut microbiome plays a pivotal role in promoting gut health in ruminants, particularly through its contributions to digestion, immune development, and long-term growth (1). Through fermentation of complex plant fibers, the microbiome extracts nutrients from sources otherwise undigestible to the host, producing metabolites, including short-chain fatty acids that support epithelial integrity and immune modulation (2, 3). Naturally, this ability makes the microbiome key to feed efficiency, where a balanced microbiome can optimize energy utilization to improve feed efficiency in beef production and reduce metabolic disorders. Moreover, the microbiome's positive effects on early immune maturation have carryover implications for animal performance, including enhanced disease resistance and sustained productivity throughout life.

The early-life microbiome is highly adaptable, undergoing rapid evolution in response to environmental cues. Factors such as transport, maternal influence, nutrition, season, and management practices profoundly shape this microbial community, influencing its diversity and functional capacity (4, 5). This fluidity makes the early-life period particularly important as, on the one hand, calves are more susceptible to pathogens, yet they also are the most amenable to microbial-led interventions. Despite these insights, most research on calf microbiomes has focused on dairy calves raised in controlled environments (6–8). Limited research has explored temporal changes in the microbiome of calves in extensive pasture-based systems, where breed diversity and environmental exposures differ markedly from intensive dairy settings. Furthermore, dairy studies predominantly examine a single breed, Holsteins, limiting generalizability due to long-term genetic selection therein (9, 10).

Increasing evidence suggests that host genetics has a significant influence on microbial trajectories and downstream productivity (11, 12). For instance, differences in gut microbial abundances have been observed between Angus and Xinjiang brown cattle, and these microbes and the metabolites they produce correlate to carcass quality traits such as intramuscular fat and backfat thickness (13). Furthermore, a multi-breed analysis of Angus, Charolais, and Kinsella beef cattle revealed correlations between rumen epithelium transcriptome and bacterial amplicon sequence variants (ASVs), underscoring the bidirectional impacts of microbial composition and epithelial function and their impact on production metrics such as feed efficiency (14).

The connections between microbial abundance and physiological traits are of particular importance in the early phase of life, where calves undergo significant changes in both their physiology and the composition of their microbiome, both of which are significant contributors to long-term health and productivity. Yet, investigation surrounding the temporal dynamics of the microbiome in beef calves remains underexplored, with few groups focusing on the first 8 weeks of life. Pasture-based beef herds often include crossbred animals selected for adaptation to local environmental and production demands. South Poll-influenced cattle are one example of an increasingly used crossbred type in forage-based systems, yet early-life gut microbiome development in calves with South Poll genetics has not been described. Yet, we lack an understanding of the microbiome in these crossbreeds, particularly among their young stock, leaving researchers and veterinarians unsure of how the microbiome is associated with the phenotypic traits these animals are selected for and unable to offer evidence-based microbial approaches.

To address these gaps, we investigated the changes in a longitudinal cohort of pasture-raised beef calves from a mixed herd composed primarily of South Poll × Angus crossbred calves. Fecal samples were collected from birth to 8 weeks of age and analyzed alongside growth performance measures to elucidate microbial evolution in extensive systems and potential links to calf productivity. We hypothesized that the calf microbiome in pasture-reared beef animals undergoes dynamic, longitudinal development during early life, reflecting combined influences of maternal environment and host genetic background.

## MATERIALS AND METHODS

### Study design and sample collection

The calves and cows studied herein came from a private pasture-raised beef herd of 100 cow–calf pairs on a private farm in southeastern Pennsylvania between April and October 2024. Cows (*n* = 7), Angus × South Poll crosses, were enrolled prior to calving. Calves (*n* = 7) were naturally delivered on pasture and enrolled sequentially. Calves consisted of three males and four females, with six crossbred animals and one Angus calf. All calves were raised together with dams as cow–calf pairs on pasture. Cow–calf pairs were segregated from the rest of the herd for the duration of the study. Animals had free access to water and mineral salt. Calves were free to nurse dams except for brief periods of separation during fecal sample collection.

Fecal samples were collected from calves and cows by rectal stimulation while in a chute. Sample collection times for calves were 1–3 days of age and weekly from 1 to 8 weeks of age, and dams were sampled once post-calving. Prior to fecal sampling, animals were weighed on a digital scale (Table 1). Fecal samples were stored in plastic deli dishes and immediately transported to the lab, where they were stored at −20°C for use until DNA extraction.

**TABLE 1:** Phenotypic characteristics of individual calves, mean ADG, mean standardized average daily gain, and changes in bodyweight as recorded across the study period^*a*^^,^^*b*^

Animal ID	Breed	Sex	24–48 h	Week 1	Week 2	Week 3	Week 4	Week 5	Week 6	Week 7	Week 8	ADG (lb/day)	Mean standardized ADG (*z*-score)
354	South Poll × Angus	Male	70.0	86.0	106.0	122.0	140.0	154.0	172.0	188.0	206.0	2.4	0.2
355	South Poll × Angus	Female	68.0	83.0	102.0	118.0	136.0	148.0	168.0	192.0	208.0	2.6	1.1
356	South Poll × Angus	Female	74.0	102.0	118.0	136.0	150.0	168.0	184.0	198.0	212.0	2.3	0.2
357	South Poll × Angus	Female	78.0	88.0	112.0	118.0	130.0	144.0	158.0	170.0	180.0	1.9	−1.8
358	South Poll × Angus	Female	72.0	100.0	114.0	130.0	146.0	162.0	172.0	194.0	212.0	2.5	0.5
359	South Poll × Angus	Male	70.0	74.0	86.0	94.0	108.0	120.0	132.0	148.0	160.0	1.7	−2.2
R384	Angus	Male	84.0	107.0	126.0	154.0	168.0	194.0	222.0	234.0	252.0	2.9	2.1

^*a*^ ADG, average daily gain.

^*b*^ Bodyweights are recorded in pounds, and ADG is expressed as pound per day across the study period.

### Extraction of genomic DNA and 16S rRNA sequencing

All fecal samples were processed to extract genomic DNA using the repeated bead beating and column method as outlined by Yu and Morrison, with added purification steps using the QIAmp Fast DNA Stool Mini Kit (15). For samples containing meconium (day 2 and week 1), only 50 mg of the sample was used to ensure a high-quality extraction, while all the other samples used 250 mg of feces. Extracted genomic DNA had the V1–V2 region of the bacterial 16S rRNA gene PCR-amplified in triplicate using the bacterial-specific primers F27 (5′-AGAGTTTGATCCTGGCTCAG-3′) and R338 (5′-TGCTGCCTCCCGTAGGAGT-3′) barcoded with a unique 12-base error-correcting Golay code for multiplexing as previously described (16). Polymerase chain reaction was performed using the Accuprime Taq DNA Polymerase System as previously described using lab protocols (7, 17) (Invitrogen, Carlsbad, CA, USA). The amplicons from each DNA sample were combined and quantified using a Quant-IT Broad Range Assay Kit, and then libraries were added to a pool in an equimolar concentration, and the pool was purified using AgencourtAMPure XP Beads (Beckman Coulter, Brea, CA, USA). Sequencing was then performed at the PennCHOP Microbiome Core using the Illumina MiSeq platform.

### Data analysis

#### Bioinformatic analysis

The bacterial 16S rRNA sequencing data were analyzed using the QIIME2 pipeline (v2020.6 [18]), as described in Hennessy et al. (7). Briefly, the sequences were separated by sample (demultiplexed) and quality filtered. ASVs were identified using the DADA2 plugin. A phylogenetic tree was built using MAFFT (19) for sequence alignment and FastTree 2 (20) for tree construction. The ASVs were classified by comparing them to the V1–V2-specific Greengenes reference database (v13.8) using a naive Bayes classifier.

Alpha diversity was calculated to measure diversity within each sample. The number of observed ASVs indicated the number of different taxa present (richness). The Shannon diversity index combined the number of taxa and their relative abundances to provide an overall estimate of diversity. Beta diversity was calculated to compare differences between samples. Weighted UniFrac accounted for the abundance of taxa, while unweighted UniFrac considered only their presence or absence. Before performing diversity analyses, the data were normalized to 3,259 reads per sample using the “qiime diversity” function.

#### Statistical analysis

All statistical analyses were performed in R (version 4.4.3). The measured alpha diversity metrics were compared between timepoints using the Wilcoxon or Kruskal–Wallis rank-sum test. The association between beta diversity matrices (weighted and unweighted UniFrac) and treatment group and timepoint was evaluated using permutational multivariate analysis of variance (PERMANOVA). As only calves were sampled longitudinally, PERMANOVA models (using the adonis2 function) were conducted on calves only, with a strata variable of Animal ID to account for repeated measures. Since sex is a sustained covariate, its *P* values would be inflated using this metric. To appropriately assess the effects of sustained covariates while accounting for within-animal temporal correlation, we first performed linear mixed-effect modeling on Unifrac distances using the model ~ timepoint as the only fixed effect. To observe the effects of sustained covariates, we used principal coordinate axes derived from Bray–Curtis dissimilarity fitting models as lmer(PCoA1 or PCoA2 ~ timepoint + sex + (1|Animal ID). Dams were included in ordination plots and descriptive summaries to visualize the progressive convergence of calf microbiota toward maternal post-calving reference communities. To evaluate the similarity between the communities of calves and adult dams, we performed pairwise PERMANOVAs on weighted and unweighted UniFrac distance matrices, comparing calves and dams as separate groups.

To identify differentially abundant genera, we employed a consensus approach incorporating multiple statistical frameworks, including linear mixed-effect modeling (lme4, lmer), Analysis of Composition of Microbiomes with Bias Correction 2 (ANCOM-BC2 [21]), and Microbiome Multivariable Associations with Linear Models (MaAsLin3 [22]), as this would reduce method-specific false positives. Genera were considered differentially abundant if they were identified as significant by at least two of the three methods. Phyla were statistically modeled using solely linear modeling. We fit linear mixed-effect models using lmer (lme4 package), with random intercepts for Animal ID. Fixed effects were Timepoint and standardized average daily gain (ADG_z), with secondary exploratory models including sex. Average daily gain (ADG) was computed per adjacent interval; for each visit up to the timepoint of 7 weeks, we used the forward interval (*t* → *t* + 1), and for week 8, our final datapoint, we used the backward interval (*t* − 1 → *t*). Consequently, the week 7 forward and week 8 backward values are identical because they refer to the same 7 → 8 interval. To obtain standardized ADG, we applied locally estimated scatter plot smoothing to fit a smooth curve to our ADG data, from which we calculated the expected ADG for each animal, the age-specific mean, and obtained the residuals for each animal. We then applied another smoothing model to the residuals to estimate the age-specific standard deviation of ADG residuals and used these to calculate ADG_z by subtracting the age-specific mean from the observed ADG and dividing by the standard deviation at that age. These calculations allowed us to determine microbiome associations with faster or slower than average daily gain relative to calves of the same age. For differential abundance testing, phyla- and genus-level relative abundances were adjusted with a pseudocount of 1, log2-transformed, and modeled using linear models. Estimates were back-transformed (2^*β*^) to obtain fold-change interpretations. This pre-specified model was used for all microbial analyses to ensure consistency. Microbial differential abundance across groups and timepoints was further assessed using ANCOM-BC2 (21) and MaAsLin3 (22), applying a prevalence filter of 0.2, and *P* values were adjusted for multiple testing using the Benjamini–Hochberg FDR correction. Interaction models were evaluated as secondary analyses; however, none were retained because they did not improve model fit and lacked biological interpretability.

To identify the “core genera,” we used genus-level count tables and associated them to construct a phyloseq object (phyloseq v1.54.0; 23). Samples from dams were excluded for calf-focused analyses. To reduce noise from very rare taxa, we applied a 0.5% relative abundance filter. Then, samples were merged by Animal ID and filtered for taxa present in ≥80% of calves. These were classified as “core taxa,” and the remaining genera in the filtered data set were considered non-core. The contribution of core taxa to community structure was quantified by calculating their combined relative abundance per sample and by comparing their contribution to microbial ecology using a distance element (Vegan package) from which we performed PERMANOVA (adonis2, vegan package) on Bray–Curtis dissimilarities with a model of the form distance ~ timepoint + sex and summed values of *R*^2^ for covariates. To generate a null distribution for non-core taxa, we performed 1,000 iterations of random sets of non-core genera of equal size to the core set without replacement and recomputed Bray–Curtis distances and PERMANOVA models. In each iteration, we again summed the *R*² values for timepoint and sex and used the mean sum to compare the distribution of summed *R*² values from non-core subsets to the core *R*². This evaluation assessed whether core genera explained significantly more age-related variance than expected by chance. For heatmap analyses, we rarefied calf fecal samples to a common sequencing depth using the phyloseq package, with a target depth equal to the minimum number of sample reads (3,259 reads). Low-abundance genera were removed following rarefaction. Genus-level abundances were averaged across animals within each timepoint to generate a single representative community profile per timepoint and converted to relative abundances. Euclidean distances between taxa were calculated, and hierarchical clustering was performed using Ward’s minimum variance method. To determine the optimal number of temporal clusters of core genera, we employed the gap statistic (R cluster package) in conjunction with k-means clustering. The optimal number of clusters (K) was chosen using the Tibshirani 1-SE rule (24), selecting the smallest K for which the observed gap value was within 1 standard error of the maximum. Heatmaps of genus counts across timepoints were generated, with rows split by the clusters of timepoints and columns defined by core genera (ComplexHeatmap package).

Microbial association networks were inferred on the core genera using SPIEC-EASI (SpiecEasi Package R [25]). We used the Meinshausen–Bühlmann neighborhood selection method, supplying the unrarefied core genus count table. The final adjacency matrix was extracted and converted to an igraph object, and clusters of tightly connected genera were identified using a community detection algorithm with igraph. Network visualizations were generated using igraph and ggplot2, with nodes colored by taxonomic group, and major modules highlighted.

Spearman correlations were calculated to test relationships between bacterial genera and animal-level traits. We used a |*ρ*| ≥ 0.40 cutoff for animal traits–genus associations and a |*ρ*| ≥ 0.20 cutoff for genus–genus associations, with Benjamini–Hochberg FDR (*q*) ≤ 0.05. For all data, differences were considered significant at *P* or *q* ≤ 0.05, and a trend was declared at 0.05 < *P* or *q* ≤ 0.10.

## RESULTS

### Changes in alpha diversity (within-sample variation) metrics with age

**Fig 1:**
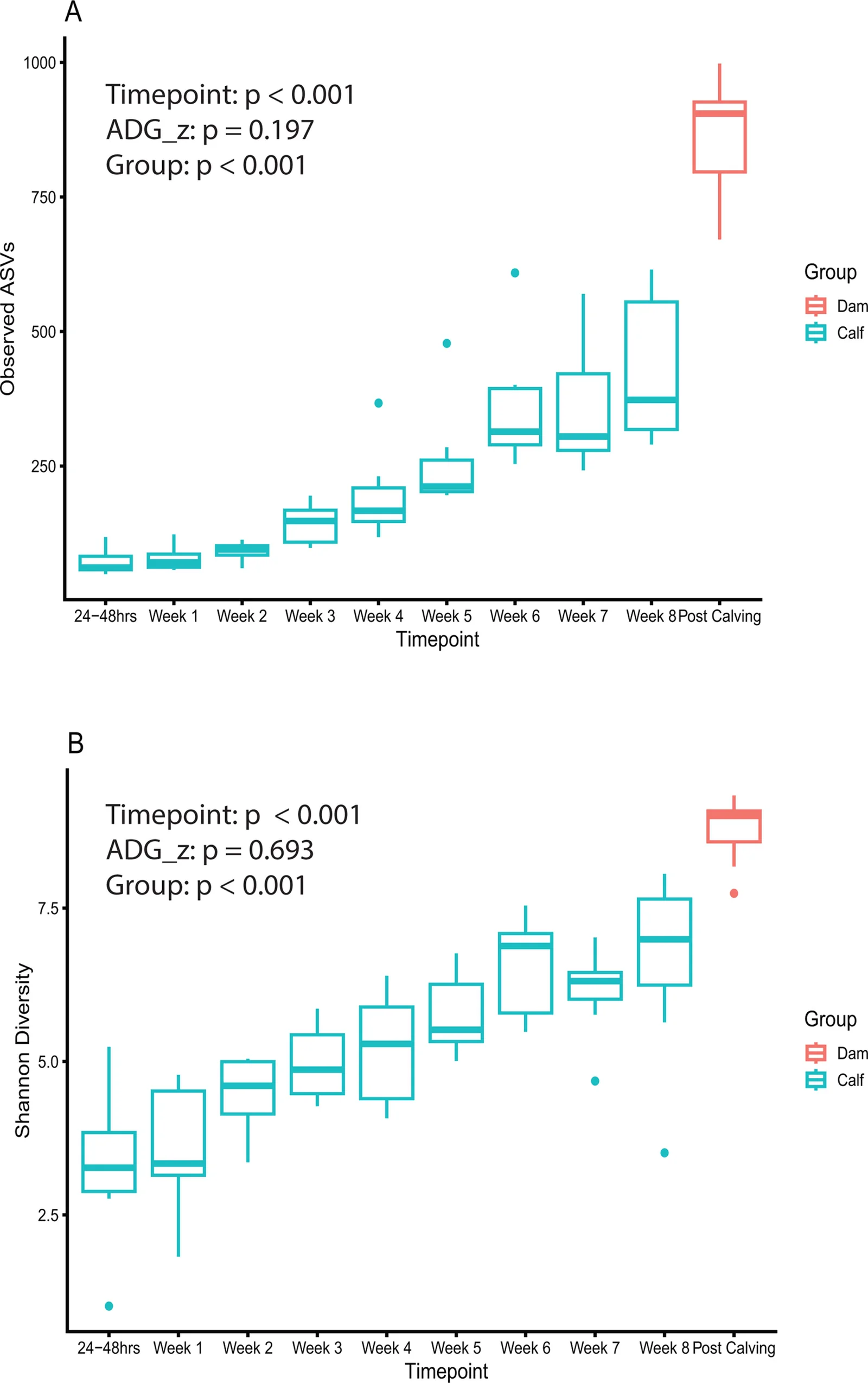
Alpha diversity of fecal bacterial communities across the study period. Data from calves (*n* = 7) and dams were grouped by age. (**A**) Species richness (observed amplicon sequence variants [ASVs]) and (**B**) Shannon diversity index. Boxes represent the interquartile range (IQR) with the median indicated by the central line and whiskers extending to 1.5 × IQR. Differences across calf timepoints were assessed using linear mixed-effect models with Animal ID included as a random effect. Pairwise comparisons among timepoints were performed using estimated marginal means with Tukey adjustment and are reported in Table S2.

Metrics for alpha diversity, or within-sample variation, included the number of bacterial species (estimated by species richness) and Shannon diversity, representing the distribution of bacterial species within each community (Fig. 1). Linear mixed modeling found a significant increase in both metrics of alpha diversity attributed to temporal changes in the microbiome (*P* < 0.001), with pairwise Tukey’s post hoc testing demonstrating significantly lower Richness and Shannon diversity in earlier timepoints (24–48 h to 2 weeks of age) compared to later ones (4–8 weeks of age; *P* < 0.001 for all comparisons to week five and beyond, *P* < 0.05 compared to week 4) (Fig. 1 and Table S2). Globally, when not controlling for fixed variables using ANOVA testing sex demonstrated a weak trend for Shannon diversity (*P* = 0.099), but this effect disappeared when using linear modeling. Standardized ADG had no significant impact on either metric.

### Comparison of fecal bacterial communities (beta diversity) by age and phenotypic factors

**Fig 2:**
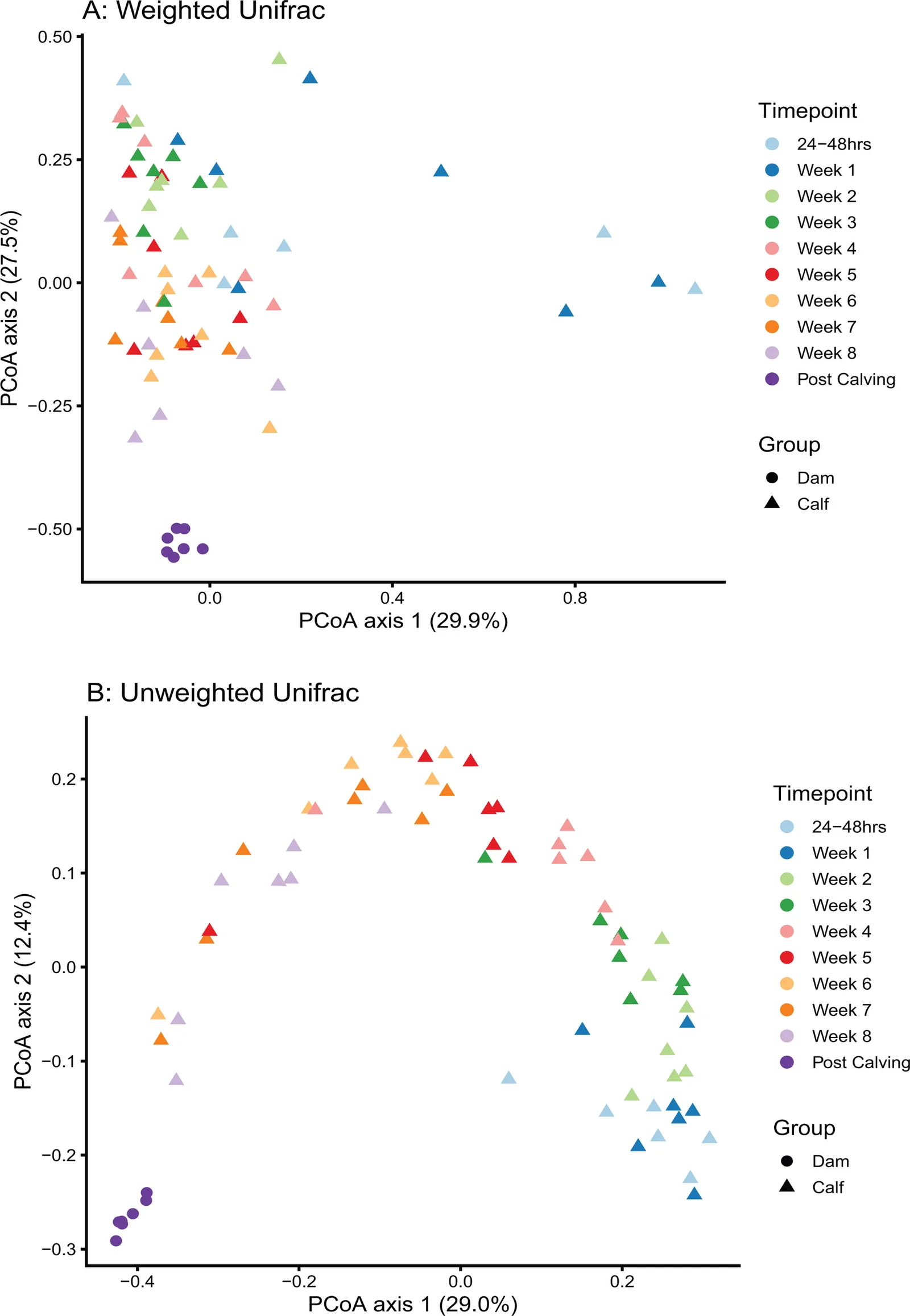
Beta diversity or between-bacterial community comparison of fecal bacterial communities by principal coordinate analysis (PCoA) across the study period. Weighted (**A**) and (**B**) unweighted UniFrac distances for calves (*n* = 7) and dams grouped by age. Differences in community structure were assessed by PERMANOVA. Fecal bacterial community structure differed significantly by age for both weighted (*R*^2^ = 0.358, *P* < 0.001) and unweighted UniFrac distances (*R*^2^ = 0.38, *P* < 0.001).

The beta diversity, or between-sample variation, of fecal bacterial communities was compared by age and phenotypic factors using adonis2 PERMANOVA models (Fig. 2). When a strata variable for Animal ID was applied to account for repeated measures, age remained significant (*P* < 0.001), while other covariates were non-significant. In linear mixed-effect models of Bray–Curtis PCoA coordinates, timepoint significantly affected axes 1 and 2 (*P* < 0.001), indicating that the primary gradient in community composition was driven by calf age. Dispersion differed between 24 h and week 1, and the other timepoints, with calves converging as they aged, indicating that differences in early timepoints may be a result of animal variance. There was also a trend in dispersion by group, as Dams were tightly clustered relative to calves.

Unweighted UniFrac results were significant by group and showed the same dispersion issues as weighted UniFrac. Distances were significant by age (*P*……) but not ADG_z for calves when strata were applied by Animal ID. When performing linear mixed-effect modeling of Bray-Curtis PCoA coordinates, again age remained the significant determinant of community structure (*P* < 0.001). In contrast, sex demonstrated a trend only for the unweighted measure of diversity along axis 2 (*P* = 0.0507), suggesting that it did not explain substantial variation in bacterial community diversity. There was a weak trend of greater dispersion among male samples and across early timepoints.

### Taxonomic composition of the calf microbiome

**Fig 3:**
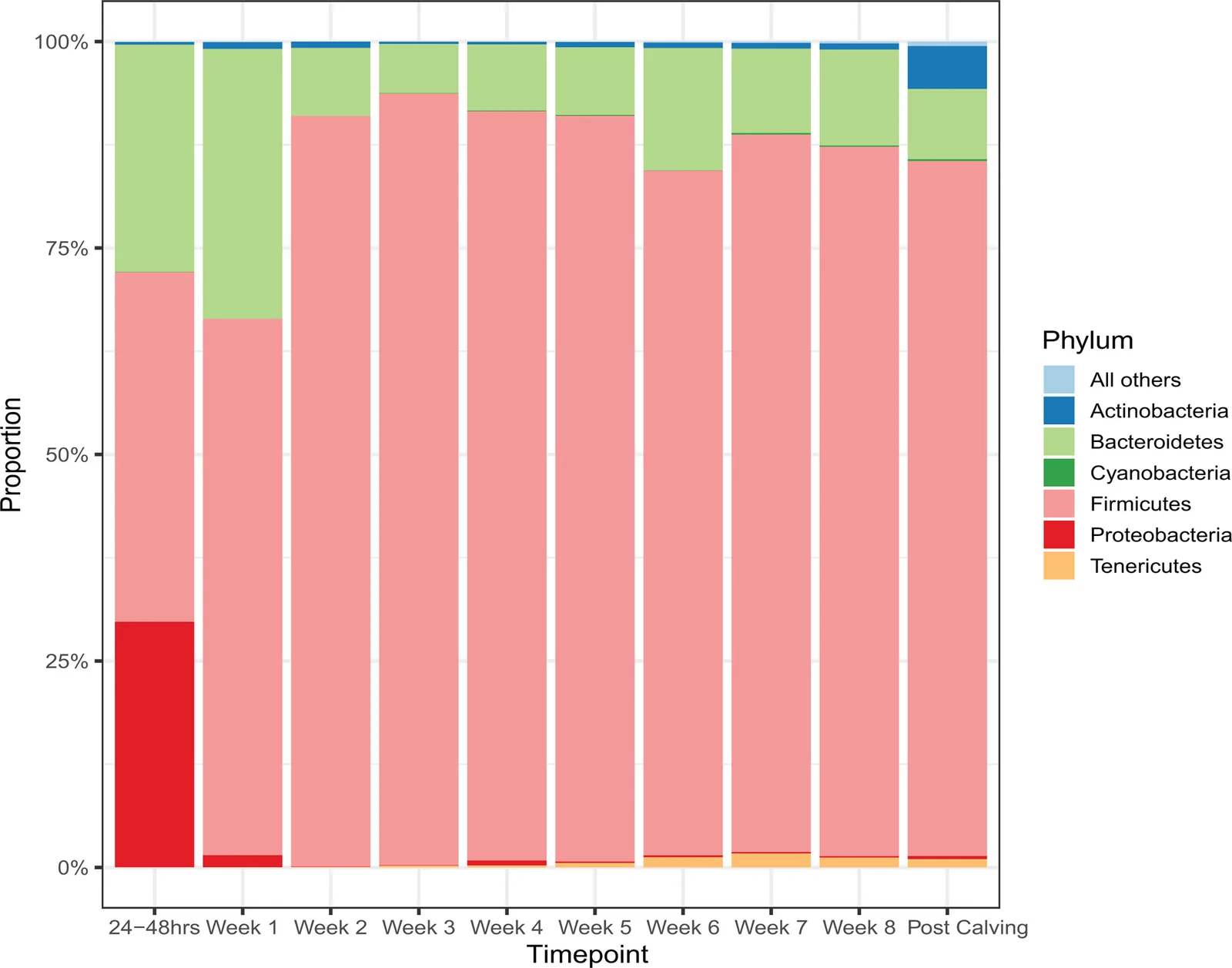
Relative abundance of dominant bacterial phyla across age. Stacked bar plots depict phylum-level composition for calves and dams grouped by sampling age.

Across fecal samples from all calves, *Firmicutes* and *Bacteroidetes* were the dominant phyla, accounting for >97% of relative abundance except for 24–48 h of age, which was marked by elevations in *Proteobacteria* and increased abundance of *Bacteroidetes* relative to *Firmicutes* (Fig. 3). There were significant increases in *Firmicutes* from week 2 onwards relative to 24–48 h and week 1, and significant increases in *Tenericutes* and *TM7* at weeks 7 and 8 relative to the period from 24 to 48 h to week 4. Moreover, there was a consistent trend of elevated *Cyanobacteria* at week 7 relative to the first three sampling timepoints. Linear modeling demonstrated a significant positive association between increasing *Actinobacteria* (*q* < 0.001) and standardized ADG. Relative to their dams, calves demonstrated significantly lower abundance of the phyla *Actinobacteria*, *TM-7*, *Spirochaetes*, *Tenericutes*, and *Cyanobacteria* (*q* < 0.05).

**Fig 4:**
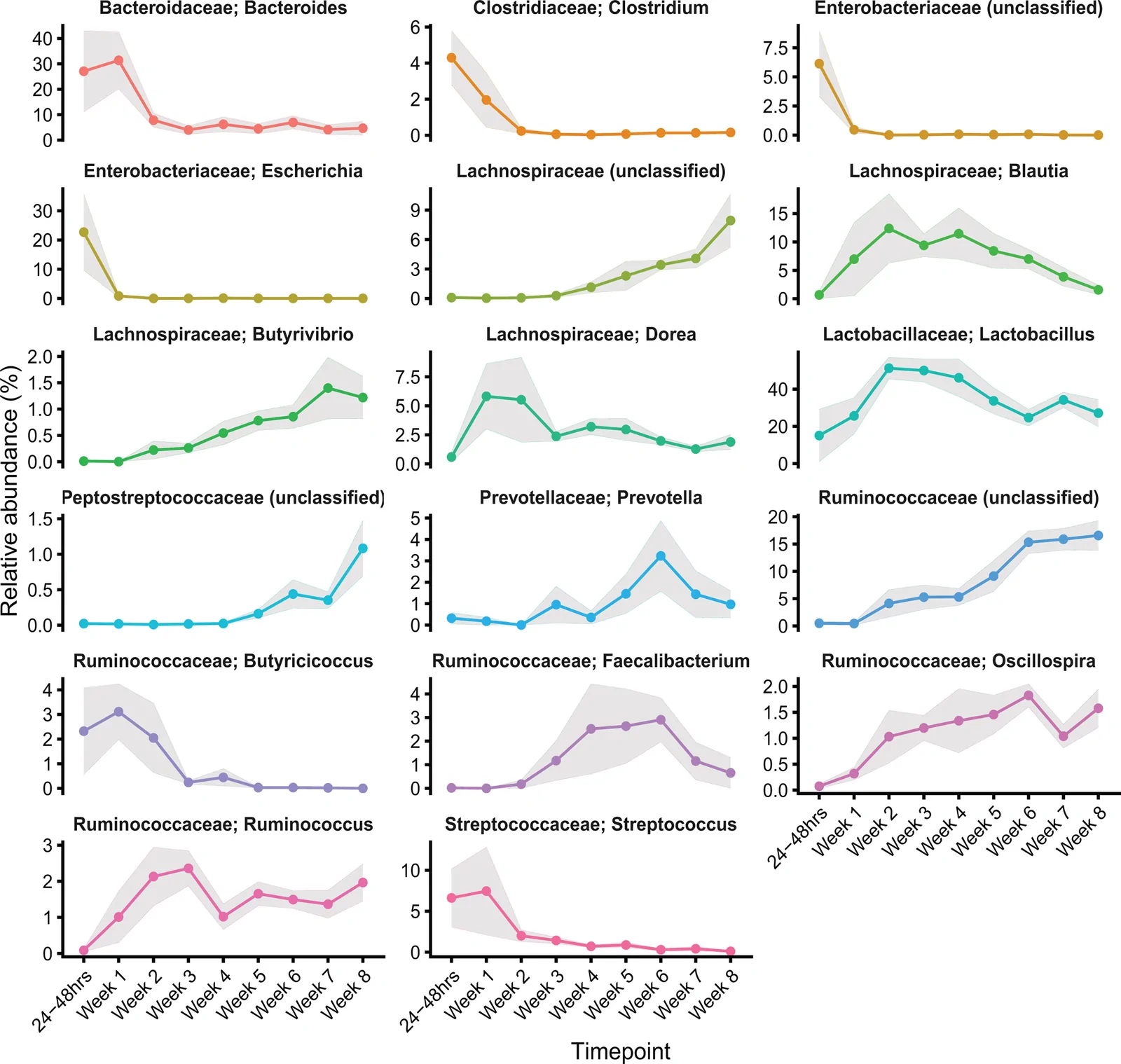
Temporal dynamics of core bacterial taxa in calves. Core taxa were defined as those present in >80% of samples with mean relative abundance >0.5%. Points and lines represent mean relative abundance across calves (*n* = 7), with ribbons indicating standard error of the mean (SEM).

We used complementary statistical approaches, including linear mixed-effect modeling, ANCOM-BC2, and MaAsLin3, to identify associations between genera and the covariates age, sex, and standardized ADG. Regardless of the approach used, age was the most significant influence on the microbial community structure (Fig. 4). Very early timepoints, including 24–48 h and week 1, were dominated by fewer than 10 genera, including *Bacteroides*, *Lactobacillus*, *Streptococcus*, *Butyricicoccus*, *Clostridium*, *Escherichia*, and unclassified members of *Enterobacteriaceae*. As calves progressed through the study, several of these genera experienced a rapid decrease in abundance, reaching a value of 0 relative to their initial abundance, including *Clostridium*, *Enterococcus*, *Escherichia*, *Staphylococcus*, *Butyricicoccus*, and unclassified *Enterobacteriaceae*. Weeks 2–5 were marked by significant increases (*q* < 0.05) in the genera *Lactobacillus*, *Blautia*, *Dorea*, *Oscillospira*, and *Ruminococcus* and a gradual decline in *Bacteroides. Lactobacillus* remained above 20% relative abundance throughout the study period, with a mean relative abundance of 37.1% from weeks 1 to 8 (Fig. 4). As calves reached the end of the study, their community structure shifted further with elevations in *Butyrivibrio* and unclassified members of the families *Lachnospiraceae*, *Ruminococcaceae*, and the order *Clostridiales* across all statistical methods. ANCOMBC2 additionally identified significant elevations in *Peptostreptococcaceae* at later timepoints.

The recurring, temporally driven successional pattern across animals suggests that the development and progression of the early microbial ecosystem is directed by a consistent set of microbes sustained across animals. Building on this observation, we followed the approach of Furman et al. and set out to define a set of microbes that would constitute a core microbiome, as defined by recurring taxa present in at least 80% of our sample cohort (26). These 25 core taxa accounted for ~94.6% of the total relative abundance per animal, and when we examined the contribution of these microbes to successional patterns, they explained substantially more community variance through all sampling timepoints (PERMANOVA *R*² = 0.423, *P* < 0.001 for timepoint and *P* < 0.05 for sex) than 1,000 permutations of randomly selected non-core genera (mean *R*² = 0.325). Looking at the succession of these core microbes across the pre-weaning period, we identified three separate temporal clusters (Fig. S3): (i) 24 h to 7 days, (ii) 2–4 weeks, and (iii) 5–8 weeks, wherein microbial abundance was matched by the statistical approaches above.

When analyzing secondary models observing timepoint in association with sex, linear mixed-effect modeling found a reduced relative abundance of *Bulleidia*, *p-75-a5*, and *Butyrivibrio*, and a strong trend for increased abundance of *Faecalibacterium* in female calves (Table S1, *q* < 0.05). MaAsLin3 demonstrated a significant decreased abundance of *Faecalibacterium* in males, an association that was descriptively observed between weeks 2 and 8, additionally identifying a trend of association for males and the increased prevalence of *Butyrivibrio*. The linear mixed-effect model revealed significant associations between the relative abundance of *Collinsella*, *Dorea*, *Streptococcus*, and *Slackia* and standardized ADG (Table S1).

Calves and dams differed significantly in the prevalence and abundance of several genera. Calf community structure was consistently marked by elevated relative abundance of *Lactobacillus*, *Bacteroides*, *Dorea*, *Parabacteroides*, and *Blautia* across all statistical methods (*q* < 0.05). Across ANCOMBC2 and MaAsLin3, dams displayed significantly higher relative abundances of unclassified *Peptostreptococcaceae* and *Coriobacteriaceae*, as well as the genus *SMB53* and *Turicibacter*, with trends for increased abundance of unclassified *Mogibacteriaceae* (*q* < 0.05).

**Fig 5:**
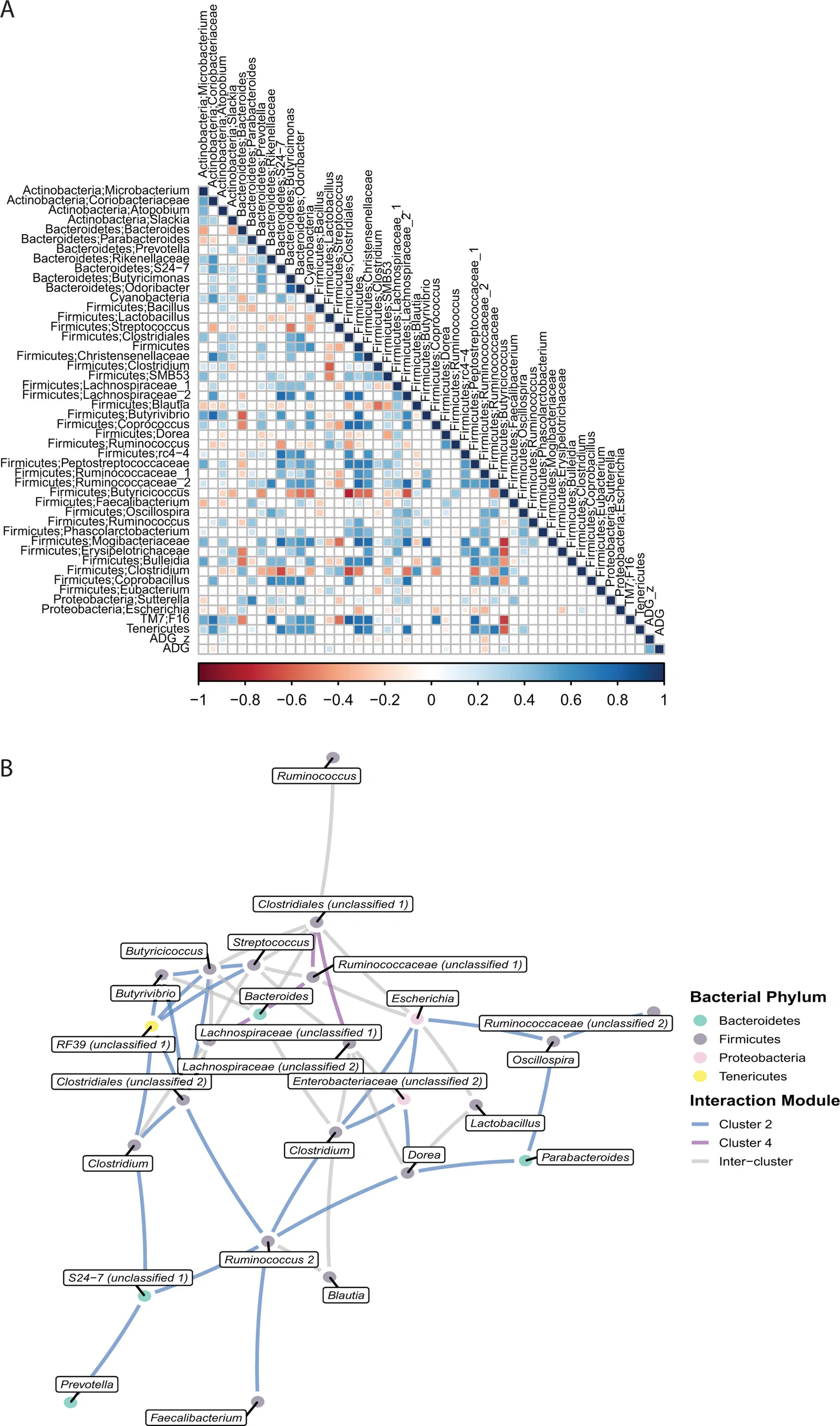
(**A**) Correlations between bacterial genera and growth metrics. Only statistically significant correlations (adjusted *P* < 0.05) are shown. Positive and negative correlations are indicated on a spectrum from navy and red, respectively. (**B**) Ecological network structure of core taxa inferred by SpiecEasi. Core taxa, those with a prevalence >80% and mean relative abundance >0.5%, were used to construct a sparse inverse covariance network. Nodes represent taxa and are colored by bacterial order. Edges represent inferred associations. Taxa were clustered using hierarchical clustering (Euclidean distance, Ward’s D2), and cluster membership (*k* = 8, determined by gap statistic) is indicated by colored intracluster edges; intercluster edges are shown in gray. Taxa are labeled by phylum, followed by the lowest available taxonomic classification.

Using Spearman correlations, we found that ADG showed weak positive correlations with *Butyricicoccus*, *Dorea*, and *Lactobacillus* (*ρ* > 0.2, *q* < 0.05; Fig. 5A). To adjust for the high collinearity of bodyweight and age, we calculated standardized ADG (ADG_Z_), which showed weak positive correlations with one taxon, *Atopobium* (*ρ* > 0.2, *q* < 0.05).

When assessing intermicrobial correlations, we focused on moderate (0.4 < *ρ* < 0.7) to strong (*ρ* > 0.7) associations among abundant taxa (Fig. 5A). Among early colonizers, *Lactobacillus* had moderate positive correlations with *Lachnospiraceae* (*Ruminococcus*) and moderate negative correlations with *Clostridium* and unclassified *Peptostreptococcaceae*. *Bacteroides* and *Streptococcus* were both moderately positively correlated with *Clostridium* (*Erysipelotrichaceae*) and negatively correlated with *Bulleidia*, *Coprococcus*, and unclassified members of *Mogibacteriaceae*, *Coriobacteriaceae*, and *Erysipelotrichaceae*. The acetogen *Blautia* showed moderate positive correlations with *Faecalibacterium*, which in turn correlated with *Parabacteroides* and *Oscillospira*. Unclassified *Ruminococcaceae* were strongly correlated with unclassified *Clostridiales* and *RF39* and moderately correlated with Mogibacteriaceae and several other taxa. However, they showed a negative correlation with taxa from the earliest sampling timepoints, including *Butyricicoccus*, *Streptococcus*, and *Clostridium*. Bacteria found at later timepoints, including *Bulleidia*, *Butyrivibrio*, and unclassified members of *Coriobacteriaceae* (*Mogibacteriaceae*) and *Peptostreptococcaceae* moderately correlated with one another (*ρ* < 0.4). Unclassified *Lachnospiraceae* and *Peptostreptococcaceae* also showed strong positive correlations with unclassified *Clostridiales* and *Mogibacteriaceae*.

With demonstrable intermicrobial correlations, we built upon our core taxa and generated a sparse conditional association network with SpiecEasi. The network was defined by five modules, two of which were dominant (Fig. 5B). Module 2, the largest, consisted of 16 of the 25 core taxa that were highly interconnected within the conditional association network, including *Streptococcus*, *Butyricicoccus*, *Butyrivibrio*, *RF39*, two species of *Clostridium*, unclassified *S24-7*, *Prevotella*, *Ruminococcus*, *Faecalibacterium*, *Escherichia*, *Dorea*, *Parabacteroides*, *Oscillospira*, and multiple unclassified *Ruminococcaceae*. Module 4 contained five taxa, including *Bacteroides*, unclassified *Ruminococcaceae*, unclassified *Clostridiales*, and two species of unclassified *Lachnospiraceae*. These modules largely recapitulated the temporal patterns observed in the heatmap (Fig. S3), with module 2 enriched in taxa colonizing from the early stage to middle stage and module 4 containing taxa that were a blend of those with a consistent presence and those from the later pre-weaning to post-weaning periods.

## DISCUSSION

The microbiome of the neonatal period has an outsized impact on the development of several organ systems in the host, including the immune, neurohormonal, and gastrointestinal systems (1). In ruminants, in particular, this developing ecosystem plays a crucial role in energy extraction and transfers to both the host gut and beyond, influencing growth and long-term productivity (2). Although extensively studied in dairy calves reared in confinement (6, 7, 26, 27), research on the microbiome in beef calves is more limited, with only a few studies focusing on the ages equivalent to the pre-weaning phase in dairy calves, from birth through 8 weeks (28–30), all using different breeds. This study characterized longitudinal changes in the fecal microbiome of purebred Angus and crossbred South Poll and Angus calf pairs co-habitating on the same pasture, revealing rapid microbial diversification and convergence toward maternal post-calving reference communities within the first 8 weeks.

Despite differences in rearing environment and breed composition, the overall trajectory of microbial maturation was comparable to that reported from our group and others for dairy calves, suggesting a conserved successional pattern (5, 7, 26, 27). Alpha diversity increased progressively with age, indicating an initial rapid colonization and expansion of ecological niches as the gut environment matured. Beta diversity analyses confirmed strong temporal clustering, with naive microbial communities at birth that gradually increased in complexity and converged with those of the dam by 8 weeks of age. Beyond differences in global metrics of diversity, we identified a conserved set of highly prevalent taxa accounting for almost 95% of the relative abundance of the fecal microbiome throughout the pre-weaning period. This core community not only dominated the microbial ecosystem but also explained substantially more of the community variance than an equally sized random set of non-core microbes, indicating that early microbial succession is principally shaped by a conserved set of taxa shared across animals. These findings reinforce the concept of a conserved, reproducible succession of taxa in the early-life microbiome, aligning with findings from the rumen (26).

The early dominance of *Lactobacillus*, *Escherichia*, *Butyricicoccus*, and other facultative anaerobes mirrors observations of dairy and beef calves and likely reflects the established colonization pattern of facultative anaerobes succeeded by generalist fermenters, such as *Bacteroides*, and subsequent development of ecological niches, a pattern seen across the mammalian family (7, 28, 30, 31). These initial populations deplete rapidly, as they are outcompeted by microbes specialized in fermenting milk or by generalist bacteria that can grow on a wide range of milk- and host-derived glycans and amino acids (32). The calves remained highly enriched in *Lactobacillus* throughout the study period, consistent with the results of Uddin et al., wherein the relative abundance of *Lactobacillus* remained above 20% in fecal samples throughout the first 60 days of life (30). In other studies, *Lactobacillus* remained highly enriched until week 4, after which its abundance fell significantly (28, 29). These differences may reflect management-related factors, including dam-rearing, continuous maternal contact, *ad libitum* milk intake, and gradual forage exposure, rather than breed-associated effects.

Interestingly, in previous dairy calf studies, *Lactobacillus* abundance in fecal samples drops rapidly after the first 1–2 weeks of life (5 ,7 , 8) or peaks around 4 weeks before falling sharply (6). Dairy calves are typically raised under controlled feeding and weaning schedules to increase their intake of concentrates and fiber. Conversely, beef calves raised on their dam are often weaned three- to fourfold later, experiencing *ad libitum* milk intake and gradually increasing forage intake as maternal milk declines (33, 34). Moreover, to reduce pathogenicity and morbidity, many dairy calves are fed milk or replacers that have undergone heat or mechanical treatment. While this reduces pathogens, it also limits the intake of commensal microbes naturally present on the teat and in milk, including skin-derived bacteria *Streptococcus*, *Propionibacterium*, and *Staphylococcus*, environmental taxa *Pseudomonas* and *Achromobacter*, and anaerobes such as *Lactobacillus*, *Bifidobacterium*, and *Corynebacterium* (35–38). Notably, the milk microbiome varies with the feeding regime, as cows on alpine pasture show enrichment in probiotic genera, such as *Lactobacillus*, *Lactococcus*, and *Bifidobacterium*, compared with those in confinement (39). Thus, constant pasture access in the dams herein may have increased milk-associated *Lactobacillus*, providing a continuous seeding of the calf gut, which would explain the sustained enrichment observed in our calves.

As calves entered week 2, we observed the development of a distinct ecology enriched in *Lactobacillus*, *Blautia*, *Dorea*, *Oscillospira*, *Ruminococcus*, *Faecalibacterium*, and *Bacteroides*, which persisted until week 5. Several moderate correlations were observed between these genera, including those between *Oscillospira*, *Ruminococcus*, *Faecalibacterium*, and *Blautia*, which also often correlated with *Parabacteroides*, although its abundance was typically lower, at around 0.5%. These key taxa comprised members of our core microbiome and may represent a broader importance as their significance has been noted in several previous studies on both dairy and beef calves (5, 7, 28–30), supporting the idea of a conserved early-life successional trajectory. In fact, temporal-based clustering of core genera further showed that these genera formed a coherent mid-successional cluster that persisted in varying degrees from weeks 2 to 6, with their dominance centered on weeks 2–5. This temporal grouping aligned closely with one of the major network modules among core taxa, indicating that this is not simply a co-occurrence but the result of integrated functional interactions and perhaps necessary syntropies, driving the fermentation of milk and resultant host development during this essential period. Moreover, *Lactobacillus*, *Bacteroides*, *Dorea*, and *Blautia* were identified as being significantly elevated in calves relative to their dams, providing further distinction to their value during this pre-weaning period in calves. This pattern is confirmed in studies on infants where *Blautia*, *Lactobacillus*, and *Bacteroides* predominate throughout infancy and decline into adulthood (40). Further investigation into this pattern and associations between these microbes could prove fruitful, generating a foundational set of microbial interventions aimed at accelerating stable colonization, enhancing growth, and reducing pathogen susceptibility during early life.

The genus *Faecalibacterium* is of considerable interest, as, along with its well-known associations with human health outcomes, several groups, including our own, have associated it with weight gain, the reduced incidence of diarrhea, and with increased intake of whole or acidified milk in calves (5, 7, 41). One species, *Faecalibacterium prausnitzii*, has had particular focus for its consistent association with epithelial health, anti-inflammation, and production of high levels of butyrate (42–44). The production of butyrate from *F. prau* uses the enzyme butyrate-CoA:acetyl-CoA transferase, necessitating an abundance of acetate, which *F. prau* does not produce. Once butyryl-CoA is made, acetate is required, as it is the molecule to which coenzyme A is transferred. This inherent requirement for acetate by *F. prau* generates a pre-condition for nearby acetogens, providing a continuous source of acetate for optimal butyrate production. Excitingly, here we saw the correlation of the acetogen *Blautia* to *Faecalibacterium*, as well as a co-localization of *Faecalibacterium* in the co-occurrence network with both acetogens, *Blautia* and *Dorea*. We previously demonstrated a similar association between *Faecalibacterium* and the acetogens *Blautia* and *Dorea* in dairy calves, indicating that this relationship may represent a conserved feature of early bovine gut microbial succession (5, 7). Similarly, the same temporal cluster of core microbes was elevated in another butyrate producer, *Oscillospira*, which instead produces butyrate using the butyrate kinase enzyme and has also been associated with feed efficiency (Gophna et al., 2018; 45). *Oscillospira* was also part of the same network module as *Faecalibacterium* but was co-localized more closely with the genus *Parabacteroides*, a succinate producer (18). Butyryl-CoA can be alternatively produced from succinate, utilizing the cofactors coenzyme A and NADPH, to form crotonyl-CoA (46). The presence of these possible syntrophies between *Faecalibacterium* and *Blautia* and between *Oscillospira* and *Parabacteroides* indicates that similar synergistic pairs within the same network module may demonstrate the functional redundancy, a prevalent phenomenon across the microbiome (47). This phenomenon applies to the early-life colonizers of beef calves, where multiple synergistic pairs work toward butyrate production. While the co-association between *Faecalibacterium* and *Blautia* has been reported in our previous report in dairy calves, *Oscillospira* and *Parabacteroides* synergy has not been reported to date and may require validation in future studies. Alternatively, the consistent co-occurrence of these relationships across bovine breeds and ruminant species suggests that these genera and their common product are not incidental but integral to the early-life microbial ecosystem and have functional importance for organ maturation, animal health, and performance.

During the third temporal clustering stage, between 5 and 8 weeks of age, there was a persistent rise in unclassified members of the *Lachnospiraceae* and *Ruminococcaceae*. This finding aligns with the current literature (26, 29, 30), underscoring the need for a deeper understanding of the heretofore unknown members of these taxa that, based on their abundance, play pivotal roles in the microbial flora of a growing calf. Progression into weeks 6–8 shows a further enrichment of unclassified *Peptostreptococcaceae*, (*Mogibacteriaceae*), *S24-7*, and *Butyrivibrio*, forming a second network of genera with strong correlations to one another and the unclassified *Lachnospiraceae*, *Clostridiales*, and *Ruminococcaceae*. These late-colonizing genera, particularly unclassified members of *Lachnospiraceae*, *Ruminococcaceae*, and *Clostridiales*, formed a distinct temporal cluster with *Butyrivibrio*, *S24-7*, and *RF39* in our heatmap analysis. This module emerged as calves increased forage intake, fitting with taxonomic findings as members such as *S24-7*, now renamed to Muribaculaceae, are associated with the degradation of long-chain dietary fibers and the production of propionate and succinate (48). These may then cross-feed to other members that are specialized in growing on smaller carbon chains, such as *RF39*, an order of bacteria deemed putative mixotrophic acetate producers (49). Interestingly, *RF39* is found in the same association network with edges connecting directly to the butyrate producer *Butyrivibrio* and other of these late-stage members, including unclassified *Clostridiales* and *Lachnospiraceae*.

Several members of these late-colonizing taxa formed a separate network module, including unclassified *Clostridiales*, and several unclassified *Lachnospiraceae* along with *Bacteroides* and *Streptococcus*, possibly indicating a “handoff,” where early colonizing generalists begin to specialize into respective niches, reducing in abundance as there is a simultaneous enrichment in a fiber-degrading consortia capable of complementing the persistent, milk-adapted *Lactobacillus*. Together, these patterns indicate a structured, late-stage successional process likely driven by fiber intake, where the early fermentative consortium, dominated by *Lactobacillus*, is gradually replaced by a later, forage-responsive, fiber-degrading consortium that expands rapidly during weeks 7 and 8 of the pre-weaning phase. The late-stage succession coincides with research on calf behavior, whereas calves entering this late pre-weaning stage are increasingly consuming substantial amounts of forage from pasture (50), especially as their nutritional requirements for growth begin to outpace the volume of maternal milk. In pasture-raised calves, increasing exploration and ingestion of forage likely creates ecological pressure favoring organisms capable of degrading complex plant polysaccharides and cross-feeding on fermentation intermediates. Thus, late-stage succession in this cohort may reflect a gradual dietary and environmental transition rather than a discrete weaning event.

Sex differences in the gut microbiome are notoriously subtle and difficult to disentangle, as genetics, diet, and age exert a strong influence on microbial composition, veiling other potential covariates (26, 51; 52). Data on this topic are sparse in ruminants, where female dairy calves represent the largest volume of research. In this study, sex-associated microbial patterns were evaluated as a secondary exploratory analysis. Minor differences were observed, including slight enrichment of *Faecalibacterium* in females, along with a trend toward increased *Butyrivibrio* in males. Both *Faecalibacterium* and *Butyrivibrio* are butyrate-associated genera, suggesting that, by taking advantage of the functional redundancy present in the microbiota, sex-related effects may result in favoring one genus over another, with no major effect on health (47). Indeed, males and females did not differ in weight, ADG, or standardized ADG, and core taxa only exhibited a weak trend of association with sex. Moreover, given the limited sample size, these patterns should be interpreted cautiously and are not presented as definitive sex effects, and larger, sex-balanced cohorts are needed to determine the meaningful contribution of sex to early community development. While this study provides a detailed temporal profile of South Poll crossed with Angus calf gut microbiome maturation, it comes with some limitations. Although the present study was limited by sample size (seven cow–calf pairs), the consistency of these trends across individuals indicates robust developmental patterns that are likely generalized to other pasture-based beef systems. Furthermore, the low sample size makes interpretation of sex-associated effects difficult; thus, findings should be considered exploratory. The primary strength of the study is its longitudinal design; however, future research building on the data herein would benefit from higher animal numbers. Pasture biodiversity and quality are geographically dependent. The localized nature of pasture grasses and rich soils of Southeastern Pennsylvania may limit the generalizability of our work to other geographical areas, although the heavy influence of milk during the pre-weaning phase may mitigate this effect. Analysis of fermentation through the production of short-chain fatty acids and gases would provide clarity on the microbiome drivers of development. Along with shotgun metagenomics, this analysis would provide a detailed breakdown of breed–pathway interactions and key microbial pathways driving differential weight gains while offering finer taxonomic detail and identifying taxa missed by our 16S analysis. Taken together, beef calves raised on pasture exhibit a reproducible, rapid development of the hindgut microbiome, facilitated by a conserved set of core genera organized into time-structured successional clusters that mirror those of dairy calves but with distinct compositional features driven by environmental and management factors.

## Data Availability

The 16S rRNA raw sequence data have been submitted to the NCBI database under BioProject accession number PRJNA1433812.

## SUPPLEMENTAL MATERIAL

Figure S1 spectrum.01730-26-s0001.eps Alpha and beta diversity stratified by sex and breed.

Figure S2 spectrum.01730-26-s0002.eps Heatmap of core taxa.

Figure S3 spectrum.01730-26-s0003.eps Relationship between raw ADG and standardized ADG.

Supplemental legends spectrum.01730-26-s0004.docx Legends for Fig. S1 to S3, Tables S1 and S2.

Table S1 spectrum.01730-26-s0005.csv Statistical linear models for genera.

Table S2 spectrum.01730-26-s0006.csv Pairwise contrasts for alpha diversity metrics by timepoint.

ASM does not own the copyrights to Supplemental Material that may be linked to, or accessed through, an article. The authors have granted ASM a non-exclusive, world-wide license to publish the Supplemental Material files. Please contact the corresponding author directly for reuse.
