# Supplementary material for: Dynamic changes in the hindgut bacterial community of pre-weaning crossbred beef calves in a pasture-based system
Source: Microbiol Spectr. 2026 Aug 21;14(10):e01730-26. doi: 10.1128/spectrum.01730-26 (PMC13688854; doi:10.1128/spectrum.01730-26)
Supplement: Supplementary file 1 [file spectrum.01730-26-s0004.docx]

**Supplementary Figure 1.** Alpha and beta diversity stratified by sex and breed. (A) Species richness and Shannon diversity (B). PCoA of (C) weighted and (D) unweighted UniFrac distances. Boxes represent IQR with medians; whiskers extend to 1.5 × IQR.

**Supplementary Figure 2.** Heatmap of core taxa (prevalence >80%, mean relative abundance >0.5%) based on rarefied counts (2850). Samples were clustered by Euclidean distance using Ward’s D2; optimal cluster number determined by gap statistic. Color scale indicates relative abundance from low (blue) to high (red).

**Supplementary Figure 3.** Relationship between raw average daily gain (ADG) and standardized average daily gain (ADG_z) across the study period. Each point represents an individual calf at a measured timepoint. The solid line represents the fitted linear regression between raw ADG and ADG_z, and shaded bands indicate 95% confidence interval around fitted relationship.

**Supplementary Table 1.** Estimated marginal means for genera modeled by timepoint, standardized average daily gain, and sex. Estimated marginal means were obtained from linear mixed-effects models fit to genus-level relative abundance data using timepoint, standardized average daily gain (ADG_z), and sex as fixed effects and AnimalID as a random intercept. Values represent model-adjusted estimates for genera across timepoints, with associated confidence intervals, p and q values and pairwise contrasts for timepoint.

**Supplementary Table 2.** Estimated marginal means with Tukey’s adjustment for multiple comparisons for Observed ASVs and Shannon Diversity. Estimated marginal means were obtained from linear mixed-effects models fit to genus-level relative abundance data using timepoint, and standardized average daily gain (ADG_z) as fixed effects and AnimalID as a random intercept. Values represent model-adjusted estimates for genera across timepoints with associated confidence intervals, p values and pairwise contrasts
